# Differential Control by 5‐HT and 5‐HT1A, 2A, 2C Receptors of Phasic and Tonic GABAA Inhibition in the Visual Thalamus

**DOI:** 10.1111/cns.12480

**Published:** 2015-11-11

**Authors:** Vincenzo Crunelli, Giuseppe Di Giovanni

**Affiliations:** ^1^ Neuroscience Division School of Bioscience Cardiff University Cardiff UK; ^2^ Department of Physiology and Biochemistry University of Malta Msida Malta

**Figure 1:**
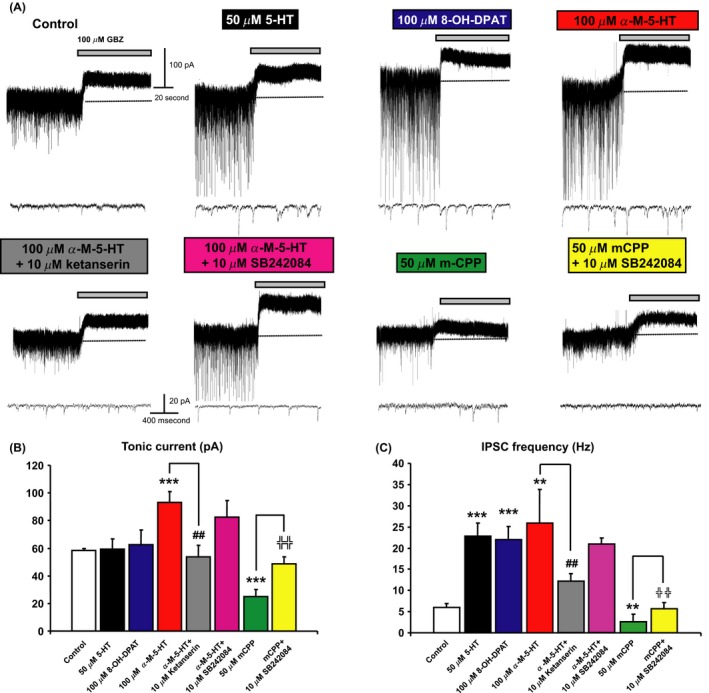
Serotoninergic modulation of phasic and tonic GABA_A_ currents in TC neurons of the dLGN. The tonic GABAA current was measured as the outward change in baseline current following focal (via a pipette) application of the GABA_A_ antagonist gabazine (100 *μ*M, GBZ, gray bar) (holding potential: −70 mV), as previously described (1). Baseline current was measured as the averaged 20 seconds current before GBZ application, while the shift in baseline current was measured as the averaged 20 seconds current after GBZ application. Focal application of GBZ (100 *μ*M) reveals different magnitude of tonic GABA_A_ current. Each 5‐HTR ligand was applied in the recording solution, either alone or in combination, and only one TC neuron was recorded in each slice. sIPSCs recorded in the different experimental conditions were collected before GBZ application and analyzed as previously described 6. (**A**) Representative current traces from different TC neurons obtained under control condition, and in continuing presence of 5‐HT (50 *μ*M, black box), 8‐OH‐DPAT (100 *μ*M, blue box), *α*‐M‐5‐HT (100 *μ*M, red box), 100 *μ*M *α*‐M‐5‐HT and 10 *μ*M ketanserin (gray box), 100 *μ*M *α*‐M‐5‐HT and 10 *μ*M SB242084 (purple box), 50 *μ*M mCPP (green box), and 50 *μ*M mCPP and 10 *μ*M SB242084 (yellow box). For each current recording (top traces), a representative pre‐GBZ period (2 seconds long, bottom traces) shows IPSCs elicited during different 5‐HT drug treatments. Summary of the effects of the different 5‐HT ligands and their combinations on tonic current (**B**) and sIPSC frequency (**C**). *P < 0.05, **P < 0.01, ***P < 0.002 versus control group. ^##^ *P* < 0.01 *α*‐M‐5‐HT + SB242084 versus *α*‐M‐5‐HT. 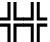 *P* < 0.01 mCPP and SB242084 versus mCPP. One‐way anova, Dunnett's multiple comparison tests. 5‐HT, serotonin; 8‐OH‐DPAT, (±)‐2‐Dipropylamino‐8‐hydroxy‐1,2,3,4‐tetrahydronaphthalene; *α*‐M‐5‐HT mCPP, meta‐chlorophenylpiperazine. sIPCS, spontaneous inhibitory postsynaptic current. SB 242084, 6‐Chloro‐2,3‐dihydro‐5‐methyl‐N‐[6‐[(2‐methyl‐3‐pyridinyl)oxy]‐3‐pyridinyl]‐1H‐indole‐1‐carboxamide hydrochloride.

Thalamocortical (TC) neurons, including those of the dorsal lateral geniculate nucleus (dLGN), one of the visual sensory thalamic nuclei, exhibit two forms of GABA_A_ receptor‐mediated inhibition: phasic or classical inhibitory postsynaptic currents (IPSCs) generated by the activation of synaptic GABA_A_ receptors (sGABA_A_R) and tonic inhibition generated by extra‐ or peri‐synaptic GABA_A_ receptors (eGABA_A_R) 1, 2. The source of GABA mediating tonic inhibition mostly arises from spillover out of the synaptic cleft, because tonic inhibition is blocked by TTX and removal of extracellular Ca2+ in adult murine dLGN TC neurons 3. Therefore, modulation of vesicular GABA release may not only affect phasic but also tonic inhibition 1, 4. Previous work in the cat and rat dLGN has shown that several neurotransmitters, including acetylcholine, serotonin (5‐HT), dopamine, and norepinephrine can modulate vesicular GABA release from inhibitory interneurons, resulting in changes in phasic inhibition (IPSC frequency), primarily through presynaptic modulation of GABA release from dendro‐dendritic synapses 5. However, except for dopamine in the somatosensory thalamus, the effect of these neurotransmitters on tonic GABA_A_ inhibition in TC neurons has not been examined. Here, we investigated whether 5‐HT and its 5‐HT_1A_, 5‐HT_2A_ and 5‐HT_2C_ receptors exert a control over tonic and phasic GABA_A_ currents in dLGN TC neurons. We used whole cell patch clamp recordings in coronal slices (300 mm) containing the dLGN from postnatal day 20–25 Wistar rats. Data analysis and experimental procedures were similar to those previously described 1, 6 and in accordance with the Animals (Scientific Procedures) Act 1986 (UK). Focal application of gabazine (GBZ, 100 mM) was used to reveal the presence of tonic GABAA current (Figure 1). All serotonergic drugs were dissolved in the recording solution, and their concentrations, co‐administration, and effects on phasic and tonic GABA_A_ current are shown in Table 1 and Figure 1. We found that 5‐HT enhances phasic GABA_A_ inhibition (i.e., spontaneous IPSCs), but has no action on tonic inhibition. These effects are identical to those observed following 5‐HT_1A/7_R activation with 8‐OH‐DPAT. On the other hand, *α*‐M‐5‐HT and mCPP enhances and reduces, respectively, both phasic and tonic GABA_A_ inhibition. These effects are dependent on 5‐HT_2A_R and 5‐HT_2C_R activation, respectively, as they are blocked by co‐perfusion with selective antagonists, ketanserin, and SB242084. Thus, the lack of 5‐HT modulation of tonic inhibition might be explained by the counterbalance of co‐activation of 5‐HT_2A_Rs and 5‐HT_2C_Rs by the endogenous ligand (Figure 1 and Table 1).

**Table 1:** Tonic and phasic GABA_A_ inhibition in dLGN TC neurons under various experimental conditions

Experimental condition	Peak amplitude (pA)	Decay time constant (*τ*_decay_, ms)	Charge transfer (fC)	Total current (pA)
Control (n = 22)	−47.0 ± 3.9	5.6 ± 0.4	−207 ± 35.5	−1.2 ± 0.3
50 *μ*M 5‐HT (n = 5)	−68.2 ± 8.6*	7.0 ± 1.1	−211 ± 126	−4.8 ± 0.7*
100 *μ*M 8‐OH‐DPAT (n = 5) *5‐HT*_*1A/7*_ *agonist*	−67.5 ± 3.4*	9.0 ± 1.5**	−157 ± 75.6	−3.4 ± 0.6*
100 *μ*M *α*‐M‐5‐HT (n = 4) *5‐HT*_*2A*_ *and 5‐HT*_*2C*_ *agonist*	−98.0 ± 8.9*	4.6 ± 1.1	−139 ± 30.3	−3.5 ± 1.1*
100 *μ*M *α*‐M‐5‐HT and 10 *μ*M Ketanserin (n = 6) *Ketanserin – 5‐HT*_*2A*_ *and 5‐HT*_*2C*_ *antagonist*	−47.3 ± 9.6	6.5 ± 1.0	−140 ± 51.5	−1.6 ± 0.4
100 *μ*M *α*‐M‐5‐HT and 10 *μ*M SB242084 (n = 6) *SB 242084 – highly selective 5‐HT*_*2C*_ *antagonist*	−64.1 ± 10.6*	5.9 ± 1.1	−164 ± 167	−3.4 ± 0.9*
50 *μ*M mCPP (n = 4) *Preferential 5‐HT*_*2C*_ *agonist*	−23.0 ± 4.5*	5.3 ± 2.1	−147 ± 80.2	−0.4 ± 0.2*
50 *μ*M mCPP and 10 *μ*M SB242084 (n = 4)	−27.6 ± 2.8*	6.2 ± 0.7*	−209 ± 21.0	−1.2 ± 0.9

Populations of individual IPSCs in a cell were averaged as described previously 6. Frequency (not listed Figure 1), peak amplitude, decay time constant (*τ*_decay_), and charge transfer of the IPSCs were measured under control condition and during drug application. Number of recorded neurons for each condition is in parentheses. Data are expressed as mean ± SD. Data were analyzed by one‐way anova (GraphPad Instat 3 software) followed by *post hoc* analyses (Dunnett's and Dunn's multiple comparison tests) *P < 0.05, **P < 0.01 versus control group.

Our findings are in agreement with recent evidence in visual cortex showing that 5‐HT enhances phasic inhibition by activating 5‐HT_2A_Rs (via calcium/calmodulin‐dependent protein kinase II, CaMKII) 7. However, whereas in visual cortex 5‐HT decreases tonic inhibition via a 5‐HT_1A_R‐dependent suppression of protein kinase A (PKA) activity) 7, we could not detect any effect on the tonic current by 5‐HT or 5‐HT_1A/7_R activation in the dLGN.

Moreover, our study is in agreement with previous *in vivo* studies in which stimulation of the dorsal raphe nucleus decreased TC neuron firing in the dLGN 8, suggesting 5‐HT had an inhibitory action. In contrast, however, *in vitro* intracellular recordings of ferret TC cells found that 5‐HT is predominantly hyperpolarizing in all thalamic nuclei tested except for the dLGN, medial geniculate and in a subset of pulvinar neurons, in which depolarizing responses were observed 9. More recently, it has been shown that in rats 5‐HT excites all TC neurons in first‐order thalamic nuclei and most (85%) TC neurons in higher order nuclei, while it hyperpolarizes the remaining cells 10. Specifically in the rat dLGN, 5‐HT_2C_R activation with *α*‐M‐5‐HT and the highly selective ligand CP‐809,101 produced depolarization of TC neurons shifting their firing from bursts to tonic 11. This would suggest that serotonin has a complex modulatory effect on thalamic nuclei which appear to be nucleus‐, 5‐HTR subtype‐, and 5‐HTR synaptic localization‐dependent. 5‐HT_1A/7_ are present in the thalamus 12, and whereas a strong 5‐HT_2C_R immunoreactivity has been detected, although not somatically, in dLGN TC neurons 13, 5‐HT_2A_R immunostaining did not reach detectable levels 11. Interestingly, both 5‐HT_2A_R and 5‐HT_2C_R mRNA are expressed in the dLGN GABAergic interneurons of young rats 14. Therefore, it is likely that the increase in sIPSC frequency that we observed in our study may result from postsynaptic 5‐HT_2_Rs on dendritic F2 terminals of dLGN interneurons, as previously shown 14.

Nevertheless, 5‐HT_1A/7_R and 5‐HT_2A/2C_Rs might also be located postsynaptically on TC neurons, and their activation may lead to phosphorylation of different subunits of sGABA_A_Rs and eGABA_A_Rs acting to differentially modulate their function. The binding of 5‐HT to 5‐HT_1A/7_R and 5‐HT_2A/2C_Rs might activate multiple signal transduction cascades, which ultimately activate different protein kinases, such as PKA or protein kinase C (PKC) and differently regulate sGABA_A_Rs and eGABA_A_Rs, as indicated by our results. On the other hand, the potential contribution of 5‐HTRs on retinal and cortical terminals can be ruled out as glutamatergic function was blocked in our preparations through the addition of kynurenic acid to the recording solution.

Overall, this study is the first to show a modulation of tonic GABA_A_ current by 5‐HTRs in the thalamus and also to highlight that phasic and tonic inhibition in the dLGN are modulated by 5‐HT through different receptor subtypes, leading to a finely tuned balance of sensory information processing in the dLGN. By showing a differential modulation of phasic versus tonic GABA_A_ inhibition, our results demonstrate a novel mechanism by which the ascending serotonergic afferents can control the thalamic gate to the visual cortex in a behavioral state‐dependent manner. Moreover, because of the putative role for thalamic tonic GABA_A_ inhibition in sleep regulation and pathological oscillations, such as those present in absence epilepsy, the opposing effects of 5‐HT_2A_Rs and 5‐HT_2C_Rs activation may provide suitable targets for pharmacological intervention in sleep and other CNS disorders.
